# Intracellular signaling prevents effective blockade of oncogenic gp130 mutants by neutralizing antibodies

**DOI:** 10.1186/1478-811X-12-14

**Published:** 2014-03-10

**Authors:** Natalie Rinis, Andrea Küster, Hildegard Schmitz-Van de Leur, Anne Mohr, Gerhard Müller-Newen

**Affiliations:** 1 Institute of Biochemistry and Molecular Biology, RWTH Aachen University, Pauwelsstraße 30, Aachen 52074, Germany

**Keywords:** Constitutively active gp130, IHCAs, Stat3, Intracellular signaling, Endocytosis, Neutralizing antibodies

## Abstract

**Background:**

Short in-frame deletions in the second extracellular domain of the cytokine receptor gp130 are the leading cause of inflammatory hepatocellular adenomas (IHCAs). The deletions render gp130 constitutively active. In this study we investigate the intracellular signaling potential of one of the most potent constitutively active gp130 mutants (CAgp130) found in IHCAs.

**Results:**

Trafficking and signaling of CAgp130 were studied in stably transfected cell lines that allowed the inducible expression of CAgp130 fused to fluorescent proteins such as YFP and mCherry. In contrast to the predominantly highly glycosylated gp130 wild type (WTgp130), CAgp130 is preferentially found in the less glycosylated high-mannose form. Accordingly, the mutated receptor is retained intracellularly and therefore less prominently expressed at the cell surface. CAgp130 persistently activates Stat3 despite the presence of the feedback inhibitor SOCS3 but fails to activate Erk1/2. *De novo* synthesized CAgp130 signals already from the ER-Golgi compartment before having reached the plasma membrane. Cell surface expressed and endocytosed CAgp130 do not significantly contribute to signaling. As a consequence, Stat3 activation through CAgp130 cannot be inhibited by neutralizing gp130 antibodies but through overexpression of a dominant-negative Stat3 mutant.

**Conclusion:**

CAgp130 and WTgp130 differ significantly with respect to glycosylation, trafficking and signaling. As a consequence of intracellular signaling pharmacological inhibition of CAgp130 will not be achieved by targeting the receptor extracellularly but by compounds that act from within the cell.

## Background

Glycoprotein 130 (gp130) is the common signal transducing receptor subunit for the interleukin (IL)-6-type cytokines. Upon stimulation with IL-6 a hexameric complex is formed comprising two molecules IL-6, IL-6Rα and gp130 respectively [1]. Janus kinases (JAKs) that are associated with the cytoplasmic part of gp130 get in close proximity and activate each other. They phosphorylate cytoplasmic tyrosine (Tyr)-residues of gp130 that serve as recruitment sites for transcription factors. There are mainly two signaling pathways activated upon IL-6 binding to gp130. The JAK/Stat pathway leads to activation of signal transducer and activator of transcription (Stat)-factors 1 and 3. These translocate into the nucleus and drive transcription of target genes like the feedback inhibitor suppressor of cytokine signaling 3 (SOCS3). The MAPK cascade gets initiated by recruitment and activation of the SH2-domain-containing tyrosine phosphatase 2 (SHP2) (reviewed in [2]).

Inflammatory hepatocellular adenomas (IHCAs) represent the most common type of hepatocellular adenoma with a frequency of 40-50% [3]. They are primarily found in women and are associated with alcohol abuse, obesity and intake of oral contraceptives. In 2009 somatic gain-of-function mutations were discovered in the *IL-6ST* gene in IHCAs coding for gp130. The resulting small in-frame deletions were found in 60% of IHCAs and are located in one of the binding sites of gp130 for IL-6. In hepatic cells these gp130 mutants caused ligand-independent Stat3 phosphorylation [4]. Two years later it was reported that 12% of IHCAs lacking a mutation in the *IL-6ST* gene harbor somatic Stat3 mutations underscoring the role of the gp130-Stat3 axis in benign hepatocellular tumorigenesis [5].

In recent years there have been numerous reports on the intracellular signaling potential of RTKs like the epidermal growth factor receptor (EGFR) and G protein-coupled receptors (GPCRs) like the β2 adrenergic receptor (β2AR) upon endocytosis (reviewed in [6]). Elaborate approaches led to the theory of signaling endosomes. Since then, spatial regulation of signal transduction has received more and more attention. Several reports focused on disease-related, mutant cytokine receptors and RTKs that show constitutive signaling [7,8].

In this study we focus on the most potent among the small in-frame deletions of gp130 found in IHCAs – del(Y186-Y190) – that result in constitutively active gp130 (CAgp130). We analyze glycosylation, cell surface expression and signaling emanating from constitutively active CAgp130. We find that CAgp130 is a potent Stat3 activator but fails to activate the MAPK cascade. Newly synthesized, intracellularly retained receptor is already able to signal. On the contrary, receptor at the plasma membrane and endocytosed receptor do not significantly contribute to constitutive activity. Our findings are of importance for potential therapeutic approaches and may contribute to treatment options for IHCAs. In a more general context CAgp130 can be used as a model system to further elucidate the interface of cancer and inflammation.

## Results

### CAgp130 exhibits deviating glycosylation and decreased cell surface expression compared to WTgp130

To analyze expression and signaling we generated HEK293 cells that allowed stable and inducible expression of differentially tagged fluorescent variants of WTgp130 and CAgp130. Utilizing the Flp-In T-Rex system and selecting single clones, cell lines were generated for expression of YFP-tagged WTgp130 and CAgp130 – T-REx-293-WTgp130-YFP and T-REx-293CAgp130-YFP respectively – as well as expression of mCherry-tagged WTgp130 and CAgp130 – T-REx-293-WTgp130-mCherry and T-REx-293-CAgp130-mCherry.

**Figure 1:**
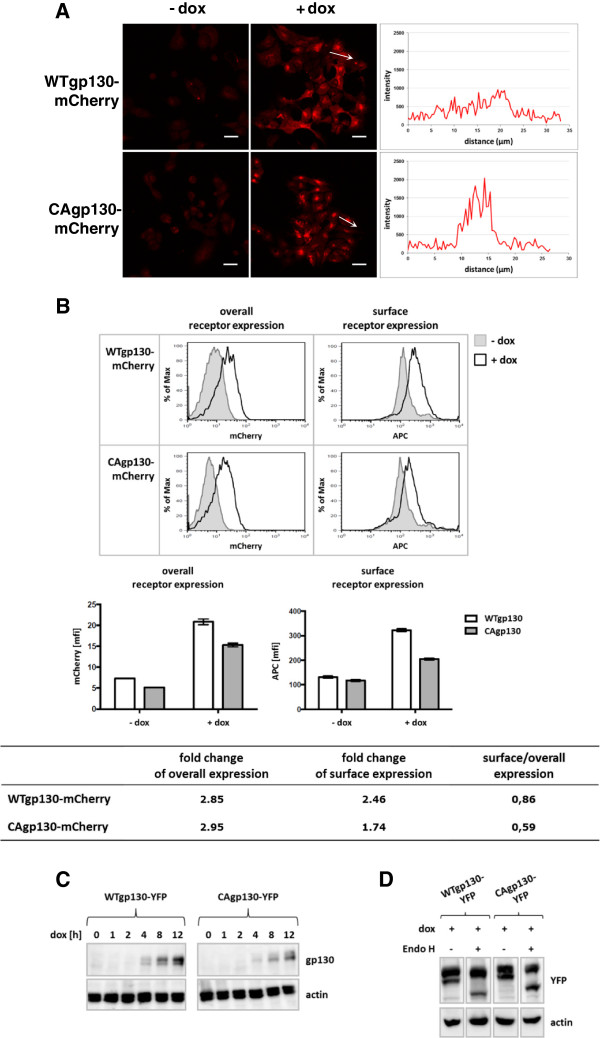
**Inducible expression of fluorescently labeled variants of WTgp130 and CAgp130. (A)** T-REx-293-WTgp130-mCherry and T-REx-293-CAgp130-mCherry were left untreated or expression was induced with 20 ng/ml dox for 48 h. Cells were fixed and receptor expression was analyzed by confocal microscopy. The diagrams represent mCherry fluorescence intensities along the length of the white arrows. Scale bars: 20 μm. **(B)** T-REx-293-WTgp130-mCherry and T-REx-293-CAgp130-mCherry were left untreated or expression was induced with 20 ng/ml dox for 24 h. Overall receptor expression was assessed by FACS analysis of the fluorescent tag (left panel) and surface receptor expression was determined through staining with the gp130 Ab B-P8 and an APC labeled secondary Ab (right panel). Non-induced cells (filled histograms) were used as negative controls. Bar charts represent means and standard deviations from three independent experiments. Fold changes in overall and surface receptor expression as well as the ratios of surface to overall receptor expression were calculated. **(C)** T-REx-293-WTgp130-YFP and T-REx-293-CAgp130-YFP were left untreated or expression was induced with 20 ng/ml dox for the indicated periods of time. TCLs were analyzed by immunoblotting using an Ab raised against a C-terminal peptide of gp130 and an actin Ab as loading control. **(D)** T-REx-293-WTgp130-YFP and T-REx-293-CAgp130-YFP were incubated with 20 ng/ml dox for 24 h. TCLs were left untreated or were subjected to endoH digestion. Subsequently, lysates were analyzed by immunoblotting using Abs against GFP/YFP and actin as loading control.

For confocal microscopy (Figure 1A) receptor expression was induced for 48 h with 20 ng/ml doxycycline (dox). Signals detected in non-treated cells are caused mainly by cellular autofluorescence. Upon induction there is a noticeable difference in the receptor distribution between cells expressing WTgp130 and CAgp130. Whereas WTgp130 is distributed throughout the cellular membrane systems the mutant CAgp130 is more concentrated in membrane structures that resemble the ER-Golgi compartment. Gp130 is known to be expressed only at very low levels at the plasma membrane [9]. Therefore, cell surface expression was analyzed by flow cytometry that is more sensitive than microscopy.

To verify total and surface receptor expression in a quantitative manner, cells stably transfected with mCherry-tagged variants of both receptors were analyzed by flow cytometry (Figure 1B). Expression was induced with 20 ng/ml dox for 24 h. Total receptor expression was assessed by the fluorescent tag. For verification of surface receptor expression non-permeabilized cells were immunostained with the gp130 antibody (Ab) B-P8 that binds to the WT and mutant receptor. Histograms in Figure 1B already point to differences between WTgp130 and CAgp130 concerning cell surface expression. Both receptors are expressed at comparable levels (left panels). However, more WTgp130 seems to reach the cell surface (right panels). Data from FACS analysis were quantified and depicted in a diagram representing the induction of overall and surface receptor expression. The table documents the reduced cell surface expression of CAgp130 that is evident from the decreased ratio of surface to overall receptor expression (Figure 1B). The same experiment performed with YFP-tagged receptors confirmed the reduced surface expression of CAgp130 (data not shown).

Verification of receptor induction by Western Blot (WB) analysis revealed detectable amounts of receptor already 4 h upon induction with 20 ng/ml dox (Figure 1C). WTgp130 is detectable as a double band that represents low and high glycosylated protein and appears mainly in the high glycosylated and fully processed form as reported previously [10]. CAgp130, however, is mainly detected in an immature form. Total cell lysates (TCLs) from both cell lines were subjected to Endo H treatment (Figure 1D). For both receptors the lower band shifted upon Endo H treatment and therefore represents the high-mannose form that has not yet completely been processed in the Golgi compartment.

### CAgp130 is a strong activator of the JAK/Stat axis but fails to activate the JAK/Erk pathway

**Figure 2:**
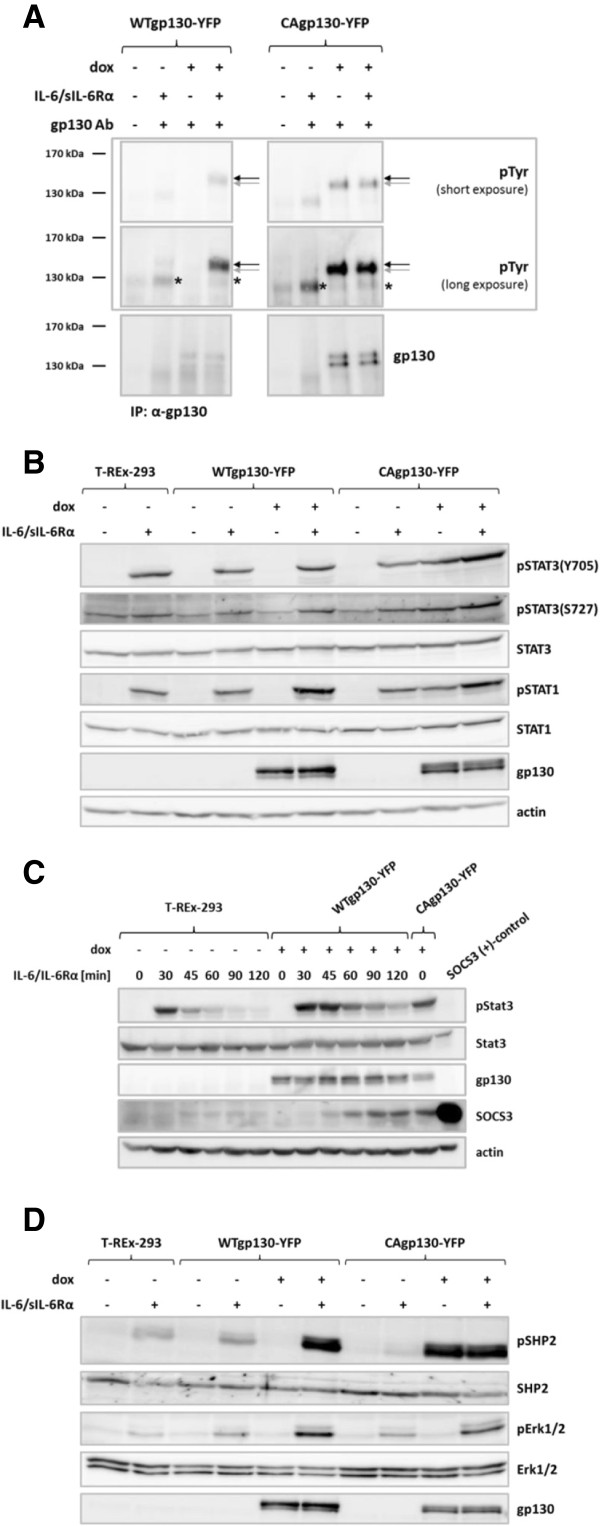
**Phosphorylation state and signaling activity of CAgp130.** T-REx-293-WTgp130-YFP and T-REx-293-CAgp130-YFP were left untreated or expression was induced with 0.5 μg/ml **(A)** or 20 ng/ml **(B, C and D)** dox for 24 h. Cells were stimulated with 200 U/ml IL-6 and 0.5 μg/ml sIL-6Rα for 15 min **(A)**, 30 min **(B and D)** or for the indicated periods of time **(C)** or left unstimulated. In **(C)** cells were puls-stimulated and the stimulus was removed after 15 min of incubation. **(A)** Gp130 was immunoprecipitated from TCLs using an antibody against the C-terminus of gp130. Precipitates were analyzed by immunoblotting using Abs against pTyr and gp130. Asterisks mark phosphorylation signal of endogenous gp130. Black and grey arrows mark the high and low glycosylated form of WTgp130-YFP and CAgp130-YFP respectively. **(B)** Activation of the JAK/Stat pathway was analyzed by immunoblotting of TCLs with Abs against pStat3(Y705), pStat3(S727), pStat1(Y701), Stat3, Stat1, gp130 and actin as loading control. **(C)** TCLs of depicted cells were analyzed by immunoblotting using Abs against pStat3(Y705), Stat3, gp130, SOCS3 and actin as loading control. For the SOCS3 positive control HEK293 cells were transiently transfected with a SOCS3 encoding plasmid. **(D)** Activation of the JAK/Erk pathway was analyzed by immunoblotting of TCLs with Abs against pSHP2, pErk1/2, SHP2, Erk1/2 and gp130.

In order to investigate signaling properties of CAgp130 and reveal possible deviations in comparison to signaling emanating from WTgp130 we first verified phosphorylation of the mutant receptor. T-REx-293-WTgp130-YFP and T-REx-293-CAgp130-YFP were incubated with dox to induce receptor expression. To stimulate phosphorylation of induced WTgp130 and endogenous gp130, samples were treated with IL-6 and sIL-6Rα as HEK293 cells do not express membrane-bound IL-6Rα. Immunoprecipitation (IP) was performed with an Ab against a C-terminal peptide of gp130 that binds to both WTgp130 and CAgp130. As can be seen in Figure 2A induced WTgp130 gets phosphorylated upon stimulation, whereas CAgp130 is phosphorylated in a ligand-independent manner. Phosphorylation of endogenous gp130 can be detected further below (marked by asterisks). For WTgp130 only the upper, fully processed form (black arrows) gets phosphorylated as it has reached the cell surface and responds to the stimulus. In the case of CAgp130, however, phosphorylation can be detected just for the lower, immature form (grey arrows). Interestingly phosphorylation of endogenous receptor is barely detectable upon induction of WTgp130 and CAgp130.

Activation of Stats was analyzed by detection of pStat3(Y705), pStat3(S727) and pStat1(Y701) (Figure 2B). Whereas WTgp130 activates Stat3 and Stat1 only upon stimulation – in the case of endogenous gp130 – or induction and stimulation – in the case of stably transfected WTgp130-YFP – CAgp130 activates both transcription factors without stimulation (Figure 2B). Furthermore we were interested to what extent CAgp130 is able to induce the feedback inhibitor SOCS3 compared to WTgp130. Parental T-REx-293 cells and T-REx-293-WTgp130-YFP were pulse-stimulated for 15 min. Upon removal of the stimulus SOCS3 expression and Stat3 phosphorylation were monitored. SOCS3 induced in the case of T-REx-293 cells was barely detectable (Figure 2C). However, SOCS3 induced by CAgp130 was detected at much higher levels that were comparable to SOCS3 triggered in cells expressing induced WTgp130 120 min after stimulation.

To verify activation of Erk downstream of JAK by CAgp130 we assessed phosphorylation of the major players SHP2 and Erk1/2. As expected, endogenous gp130 can activate SHP2 and Erk only upon stimulation. In cells additionally expressing WTgp130 as a YFP-tagged protein activation is stronger upon induction as far more receptor molecules are available (Figure 2D). Surprisingly there is just a partial activation of the JAK/Erk axis by CAgp130. Upon induction of mutant receptor SHP2 gets heavily phosphorylated. However, there is hardly any activation of Erk1/2 detectable. Activation of the JAK/Erk cascade by CAgp130 seems to be strictly limited. Similar observations were made with untagged receptor (data not shown). No activation of Akt above background levels was detectable in HEK cells expressing CAgp130 (data not shown).

### WTgp130 and CAgp130 show different functionality of cytoplasmic Tyr-residues

Previous work by Stahl et al. [11] and Gerhartz et al. [12] has pointed out the importance of individual pTyr motifs for activation of specific Stat proteins. Using these pTyr motifs the last four cytoplasmic Tyr-residues were identified as recruitment sites for Stat3 within the consensus sequence YXXQ. Stat1 was found to be recruited to the two most distal cytoplasmic Tyr-residues of gp130 and to the more restricted consensus YXPQ. Work of Schmitz et al. [13] additionally demonstrated differential contribution of potential recruitment sites for Stat3 activation.

**Figure 3:**
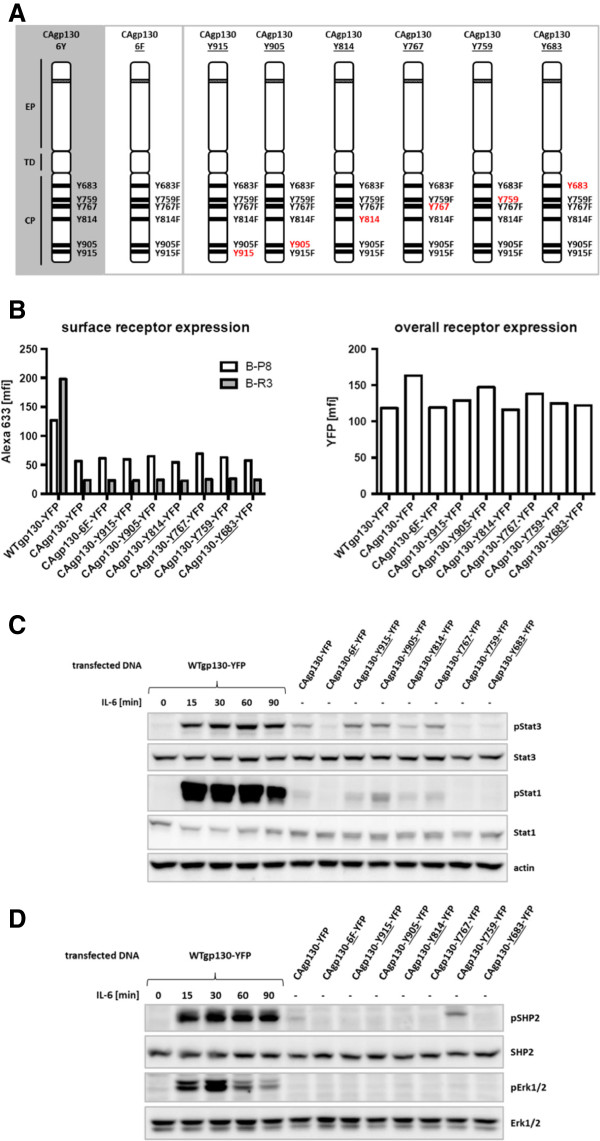
**Functional analysis of individual cytoplasmic Tyr-residues of CAgp130. (A)** Schematic overview of add-back mutants of CAgp130. EP: extracellular part with depicted del(Y186-Y190), TD: transmembrane domain, CP: cytoplasmic part. HEK293 cells stably expressing IL-6Rα were transiently transfected with WTgp130-YFP, CAgp130-YFP, CAgp130-6F-YFP or YFP-tagged add-back mutants of CAgp130. **(B)** Overall receptor expression was assessed by FACS analysis of the fluorescent tag (right panel). Surface receptor expression was verified using the gp130 Abs B-P8 and B-R3 and an Alexa633 labeled secondary Ab (left panel). **(C)** and **(D)** Cells were stimulated with 200 U/ml IL-6 for the indicated periods of time or left untreated. TCLs were analyzed by immunoblotting. **(C)** Activation of the JAK/Stat pathway was verified by Abs against pStat3(Y705), pStat1(Y701), Stat3, Stat1 and actin as loading control. **(D)** JAK/Erk pathway activation was assessed using Abs against pSHP2, pErk1/2, SHP2 and Erk1/2.

In order to define the contribution of cytoplasmic Tyr-residues of CAgp130 for activation of Stat proteins and SHP2 we generated a series of so-called add-back mutants of CAgp130, where just single cytoplasmic Tyr-residues are available for signaling (Figure 3A). Furthermore a mutant of CAgp130 without any cytoplasmic Tyr-residues was generated – CAgp130-6F-YFP – to serve as a negative control. Constructs encoding WTgp130-YFP, CAgp130-YFP, CAgp130-6F-YFP and add-back constructs were transiently transfected in HEK cells stably expressing IL-6Rα. Transfected cells were subjected to FACS analysis to verify overall and surface expression of the mutants (Figure 3B). Overall receptor expression was assessed using the YFP tag and surface receptor was stained by two different monoclonal Abs targeting distinct sites on the extracellular part of gp130. Ab B-P8 targets domain 3 (D3) of the extracellular part of gp130 and detects both WTgp130 and CAgp130. Ab B-R3 targets D2 of gp130 and does not detect CAgp130 probably because of the activating deletion located within this domain. FACS analysis using Ab B-P8 reveals a considerably increased amount of surface WTgp130 compared to CAgp130 in agreement with the FACS data shown in Figure 1. CAgp130-6F-YFP without any cytoplasmic Tyr-residue and the series of add-back mutants do not show any difference in surface expression compared to CAgp130 indicating that single Tyr-residues do not have any impact on cell surface expression.

To study effector functions of single pTyr-residues of CAgp130 on the JAK/Stat axis TCLs were probed for pStat3(Y705) and pStat1(Y701). As shown in Figure 3C there are four cytoplasmic Tyr-residues that are able to bind Stat3 and Stat1 upon phosphorylation. Activation of Stat3 by CAgp130 exclusively occurs through the four distal Tyr-residues in line with findings for WTgp130 [12]. The two distal Tyr-residues seem to be favored as they lead to stronger Stat3 activation than the two membrane-proximal ones. Stat1 gets also activated through binding to the four distal Tyr-residues with the second to last pTyr being the most preferred activation site. STAT activation through the add-back mutants is stronger than through CAgp130-YFP harboring all Tyr-residues. This might be a consequence of the fact that the STAT-activating add-back mutants lack Y759 required for feedback inhibition through SOCS3. Thus, CAgp130-YFP is to a certain extent sensitive to feedback inhibition. Accordingly, upon strong overexpression of SOCS3 signaling of CAgp130 ceases (data not shown and [14]).

With respect to activation of the JAK/Erk cascade TCLs of cells transfected with add-back mutants were probed for SHP2 and Erk phosphorylation (Figure 3D). In line with results shown in Figure 2D phosphorylation of SHP2 but not Erk can be detected in cells transfected with CAgp130. Activation of SHP2 caused by CAgp130 can be definitely assigned to the second Tyr-residue proximal to the membrane – Y759 – in line with published data [11]. In cells transfected with the CAgp130 that only harbors the SHP2 recruitment site SHP2 activation is even stronger than in cells expressing CAgp130, still there is no Erk phosphorylation detectable.

### *De novo* synthesized CAgp130 is able to signal from intracellular compartments before reaching the cell surface

**Figure 4:**
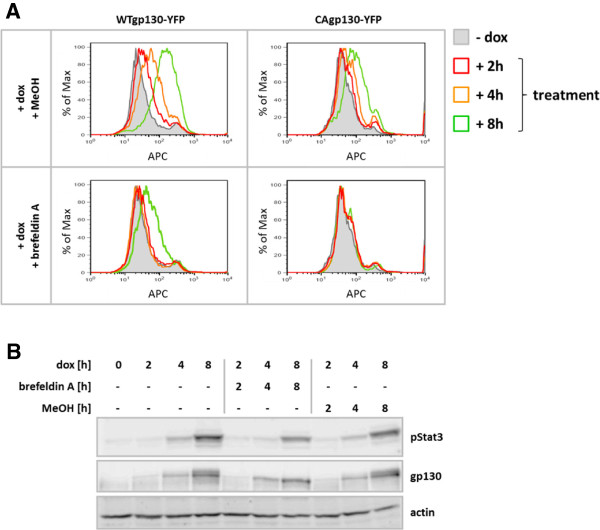
**Effect on signaling by intracellular retention of *de novo *synthesized CAgp130.** T-REx-293-WTgp130-YFP and T-REx-293-CAgp130-YFP were left untreated or expression was induced with 20 ng/ml dox for the indicated periods of time. Cells were simultaneously treated with 100 ng/ml brefeldin A or MeOH (vehicle). **(A)** Overall receptor expression was assessed by FACS analysis of the fluorescent tag (Additional file 1) and surface receptor expression was determined through staining with the gp130 Ab B-P8 and an APC labeled secondary Ab. Non-induced cells (filled histograms) were used as negative controls. **(B)** TCLs were analyzed by immunoblotting using Abs against pStat3(Y705), gp130 and actin as loading control.

In order to investigate whether signaling of CAgp130 is dependent on its localization at the cell surface T-REx-293-WTgp130-YFP and T-REx-293-CAgp130-YFP were treated with dox to induce receptor expression. Simultaneously cells were treated with 100 ng/ml brefeldin A to prevent newly synthesized receptor from reaching the cell surface. Cells were analyzed by flow cytometry. Overall expression of the receptor was assessed by the YFP tag (Additional file 1) and cell surface receptor was detected by the gp130 Ab B-P8 and an APC labeled secondary Ab. As shown in Figure 4A dox treatment leads to the increase of receptor surface expression for both WTgp130 and CAgp130 with less CAgp130 reaching the plasma membrane. This increase is already detectable upon 4 h of induction. The combination of induction and treatment with brefeldin A causes complete retention of WTgp130 for the first 4 h. According to the FACS analysis at the 8 h time point a small amount of WTgp130 escapes retention and appears on the cell surface. In the case of CAgp130 retention seems to be more efficient probably due to the smaller amount of receptor that reach the plasma membrane at all. Brefeldin A in the applied concentration is able to completely retain CAgp130 within the cell even 8 h after induction. A considerable amount of surface receptor is detectable upon 8 h of induction in the vehicle control for CAgp130.

TCLs of T-REx-293-CAgp130-YFP were subjected to WB analysis and probed for CAgp130 expression and Stat3 phosphorylation (Figure 4B). Upon induction increasing amounts of CAgp130 and stimulus-independent Stat3 phosphorylation can be detected. Upon treatment with brefeldin A the upper, higher glycosylated receptor band disappears. Thus, retention of CAgp130 and generation of an ER-Golgi hybrid compartment prevent complete glycosylation of the receptor. Nonetheless, the retained receptor is still able to phosphorylate Stat3 from within the cell.

### Capturing CAgp130 at the cell surface does not markedly influence its signaling activity

After having assessed activity of *de novo* synthesized, intracellularly retained CAgp130 we further tried to elucidate whether mutant receptor is able to signal from the plasma membrane or intracellular compartments upon endocytosis.

In previous work Thiel et al. [15] reported that gp130 undergoes stimulus-independent internalization and that it is constitutively associated with the AP-2 adaptor complex. These findings suggest that gp130 is constitutively endocytosed in a clathrin- and therefore dynamin-dependent way.

In order to elucidate whether endocytosis of CAgp130 is dependent on dynamin we utilized the dominant-negative K44A dynamin mutant [16]. To be able to distinguish non-transfected cells from cells transfected with dynamin, a construct was generated that allows simultaneous expression of K44A dynamin and GFP separated by an internal ribosomal entry site (IRES) – K44Adynamin/GFP.

**Figure 5:**
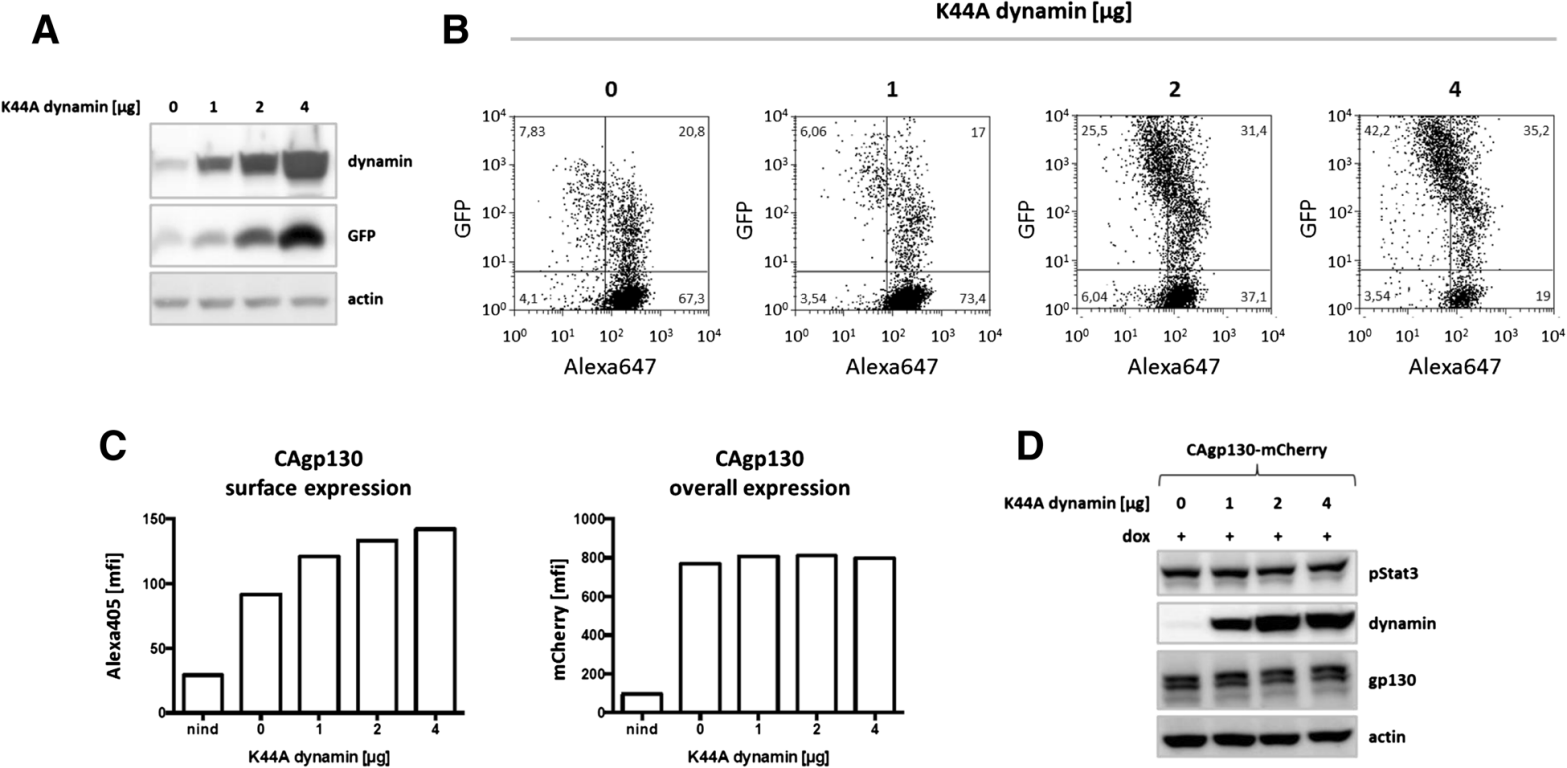
**Effect of dominant-negative dynamin on surface expression and signaling of CAgp130. (A)** and **(B)** T-REx-293 cells were transiently transfected with increasing amounts of an expression vector encoding dominant-negative K44A dynamin and GFP. **(A)** TCLs were analyzed by immunoblotting using Abs against dynamin, GFP and actin as loading control. **(B)** Cells were incubated with Alexa647 labeled transferrin. K44A dynamin expression and transferrin uptake were assessed via FACS analysis. **(C)** and **(D)** T-REx-293-CAgp130-mCherry were transfected with increasing amounts of dominant-negative K44A dynamin. Cells were left untreated or expression of CAgp130 was induced with 20 ng/ml dox for 24 h. **(C)** Overall receptor expression was assessed by FACS analysis of the fluorescent tag (right panel) and surface receptor expression was verified using the gp130 Ab B-P8 and an Alexa405 labeled secondary Ab (left panel). **(D)** TCLs were analyzed by immunoblotting using Abs against pStat3(Y705), dynamin, gp130 and actin as loading control.

Initially, we tested functionality of dominant-negative dynamin by verification of its inhibitory effect on the dynamin-dependent transferrin uptake in T-REx 293 cells. T-REx 293 cells were transfected with increasing amounts of K44Adynamin/GFP and incubated with Alexa647 labeled human transferrin. Figure 5A shows concomitant increase in dynamin and GFP signals upon transfection of increasing amounts of K44Adynamin/GFP. Transfected cells were analyzed by flow cytometry. As shown in Figure 5B with transfection of increasing amounts of K44Adynamin/GFP more and more counted cells shift to the GFP^+^ population and show reduced Alexa647 fluorescence indicating a reduction in transferrin uptake. Cells not transfected with dynamin were transfected with a construct encoding IRES-GFP in order to have a GFP^+^ population where transferrin uptake is not perturbed.

Next, cells inducibly expressing CAgp130-mCherry were transfected with increasing amounts of K44Adynamin/GFP. About 24 h after transfection cells were treated with dox for 24 h and subsequently analyzed by flow cytometry. GFP^+^ and therefore dynamin transfected cells were analyzed with respect to overall and surface receptor expression. Overall receptor expression was verified via the mCherry tag and surface receptor was monitored using the gp130 Ab B-P8 and an Alexa405 labeled secondary Ab. As shown in Figure 5C overall receptor expression is not affected by transfection of dominant-negative dynamin. Non-induced cells serve as a negative control. On the contrary, the amount of cell surface receptor increases with transfection of increasing amounts of K44Adynamin/GFP. This result indicates that CAgp130 gets internalized in a dynamin-dependent way.

To find out whether inhibiting receptor endocytosis has any effect on signaling of CAgp130 TCLs of cells transfected with increasing amounts of K44Adynamin/GFP were subjected to WB analysis and probed for pStat3 (Figure 5D). Surprisingly, inhibition of endocytosis does not seem to have any effect on signaling. This result implies that receptor at the cell surface and receptor molecules upon endocytosis do not considerably contribute to signaling of CAgp130 if they contribute at all.

### Neutralizing gp130 Abs do not impair constitutive activity of mutant receptor

In order to further substantiate the finding that cell surface as well as endocytosed receptor molecules do not essentially contribute to the constitutive activity of CAgp130 we tried to inhibit mutant receptor with antagonistic gp130 Abs. The applied Abs used in this study were developed in previous work by Wijdenes et al. [17] to inhibit the biological activity of distinct IL-6-type cytokines through gp130. Taking into account the recent publication by Sommer et al. [18] where CAgp130 was reported to be inhibited by a gp130 Ab that specifically neutralizes IL-11 signaling, we included the referred Ab B-P4 in our study. In addition we utilized gp130 Abs B-T2 and B-R3. B-T2 was originally shown to downregulate IL-6 induced signaling and proliferation of a human myeloma cell line. B-R3 was shown to downregulate IL-6 as well as IL-11 induced signaling. As mentioned before B-R3 targets domain D2 of gp130 and is not able to bind to CAgp130. Thus it serves in the context of the mutant receptor as a negative control.

**Figure 6:**
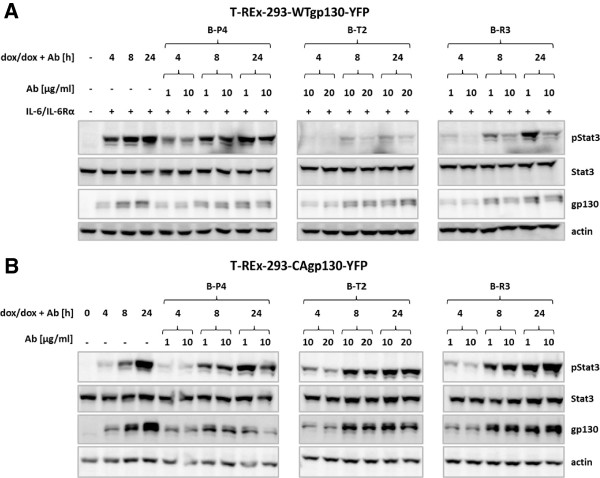
**Effect of neutralizing gp130 Abs on signaling of CAgp130.** T-REx-293-WTgp130-YFP **(A)** and T-REx-293-CAgp130-YFP **(B)** were left untreated or expression was induced with 20 ng/ml dox for the indicated periods of time. Cells were simultaneously incubated with indicated amounts of neutralizing gp130 Abs and subsequently stimulated with 200 U/ml IL-6 and 0.5 μg/ml sIL-6Rα or left unstimulated. TCLs were analyzed by immunoblotting using Abs against pStat3(Y705), Stat3, gp130 and actin as loading control.

T-REx-293-WTgp130-YFP and T-REx-293-CAgp130-YFP were treated with dox to induce receptor expression and were left untreated or were incubated with the given concentrations of Abs B-P4, B-T2 or B-R3. In order to analyze the inhibitory effect on WTgp130 expressing cells stimulation was performed with IL-6 and sIL-6Rα. Binding of the Abs was verified by FACS analysis using an APC-tagged secondary Ab (Additional file 2). TCLs were subjected to WB analysis and probed for Stat3 phosphorylation (Figure 6A,B). As shown in Figure 6A IL-6 induced Stat3 phosphorylation can be inhibited by Abs B-T2 and B-R3 and to some extent with Ab B-P4 in a dose- and time-dependent manner. Strikingly there is no effect of any of the neutralizing Abs on Stat3 phosphorylation caused by CAgp130 (Figure 6B).

### Dominant-negative Stat3-Y705F interferes with constitutive activity of CAgp130

**Figure 7:**
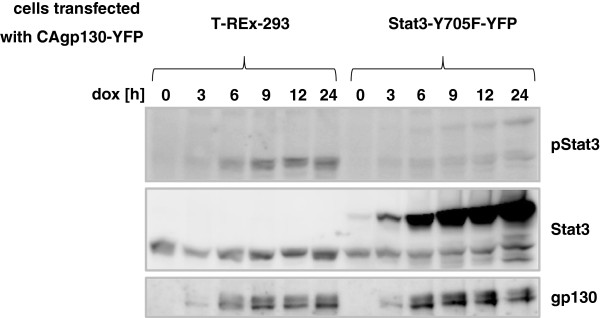
**Effect of dominant-negative Stat3 on signaling of CAgp130.** T-REx-293 cells and cells stably transfected with Stat3-Y705F-YFP were transfected with equal amounts of CAgp130-YFP. Expression of CAgp130 or CAgp130 and Stat3-Y705F was induced with 20 ng/ml dox for the indicated periods of time. TCLs were analyzed by immunoblotting using Abs against pStat3(Y705), Stat3 and gp130. The detected endogenous Stat3 serves as loading control.

In order to downregulate constitutive Stat3 phosphorylation caused by CAgp130 from within the cell we took advantage of the dominant-negative Stat3-Y705F mutant. Stat3-Y705F impairs WT-Stat3 activity in stimulated cells and was recently reported to act at multiple levels affecting phosphorylation, nuclear translocation and transcriptional activity of WT-Stat3 upon stimulation [19]. Parental T-REx-293 cells and cells inducibly expressing Stat3-Y705F-YFP were transfected with equal amounts of CAgp130-YFP. Upon induction there is an increase in expression of CAgp130 and ligand-independent Stat3 phosphorylation in T-REx-293 cells over time (Figure 7). In cells stably transfected with dominant-negative Stat3, expression of transiently transfected CAgp130 as well as Stat3-Y705F-YFP is induced upon dox treatment. Stat3-Y705F-YFP strongly attenuates CAgp130-mediated phosphorylation of endogenous Stat3.

## Discussion

In this study we focused on the intracellular signaling activity of CAgp130. We report that *de novo* synthesized mutant receptor is able to signal on its way to the plasma membrane and that neither plasma membrane receptor nor endocytosed receptor significantly contribute to constitutive activity. Among the most striking characteristics of CAgp130 are deviations in glycosylation and subcellular distribution compared to WTgp130. The mutant receptor is mainly present in the immature, high-mannose form and resides at intracellular membranes.

Similar studies have already been performed for a constitutively active mutant of the thrombopoietin receptor MPL [7], as well as a series of receptor tyrosine kinases (RTKs) like FLT3-ITD [20] and constitutively active Kit [21]. Defects on glycoprotein maturation are coupled to the ER quality control (reviewed in [22]). Incorrectly folded glycoproteins interact with ER chaperones and this interaction causes retention within the ER. While this manuscript was in preparation Schmidt-Arras et al. reported that ER retention of CAgp130 is mediated by its interaction with the ER chaperone calnexin confirming this assumption [23]. Similar studies revealed the interaction of calnexin with FLT3-ITD, a RTK that was also reported to show incomplete glycosylation and impaired cell surface expression [20]. However, in the case of FLT3-ITD and several other RTKs inefficient maturation is rather due to constitutive kinase activity and tyrosine phosphorylation than defective glycosylation. From our results it is obvious that this is not the case for CAgp130 as a mutant where all cytoplasmic residues have been replaced shows unaltered surface expression compared to CAgp130. Furthermore retention of CAgp130 does not activate the unfolded protein response (UPR) [23] – a stress response initiated by the accumulation of unfolded or misfolded proteins in the ER [reviewed in [24]). This report is in line with our findings that show no induction of the chaperone binding immunoglobulin protein (BiP) upon strong induction of receptor expression (data not shown). In addition we can confirm that CAgp130 is not primarily degraded by the proteasome and therefore exclude ER associated degradation (ERAD) [22]. Preliminary data indicate stabilization of CAgp130 in the presence of lysosomal inhibitors (data not shown).

Apart from processing and subcellular distribution we found further differences between CAgp130 and WTgp130 concerning their signaling activity. The mutant receptor strongly activates Stat3 and induces the feedback inhibitor SOCS3, however, it only causes partial activation of the JAK/Erk cascade. Although SHP2 gets phosphorylated in a ligand-independent manner there is no Erk activation detectable. A possible explanation for this fact is based on the limited spatial availability of components of the MAPK cascade at intracellular membranes. The adaptor protein Gab1 is necessary for activation of the MAPK cascade upon stimulation with several cytokines such as IL-6 and EGF. Gab1 gets recruited to the plasma membrane via its PH-domain and this recruitment was reported to be mandatory for its activation [25], making activation of the JAK/Erk cascade to a process strictly limited to the plasma membrane. This finding in combination with the low receptor amount on the cell surface can possibly explain our unexpected results. Similar observations on spatial regulation of receptor activity were made in the case of FLT3-ITD [8]. Targeting of FLT3-ITD to the plasma membrane actually reversed its signaling activity strongly activating MAPK and PI3K pathways and diminishing Stat5 activation. Taken together these data point out major deviations in the processing-trafficking-signaling axis between CAgp130 and WTgp130. These differences can be attributed to an intrinsic glycosylation defect and do not rely on constitutive phosphorylation.

In order to find out whether CAgp130 shows any intracellular activity we utilized the pharmacological inhibitor brefeldin A. Here we report that newly synthesized CAgp130 activates Stat3 before reaching the plasma membrane, in line with recently published data [23]. This observation is further in line with the finding that only immature receptor gets phosphorylated in the case of CAgp130. The observed decrease in Stat3 phosphorylation correlates with the reduction of overall receptor amount. Another possible explanation would be steric hindrance of receptors accumulating in the brefeldin-induced ER-Golgi compartment. Yet a further interesting scenario would be that receptors at intracellular membranes are less potent in activating signaling pathways than receptors at the plasma membrane, bringing up the spatial regulation of receptor activity. Stat3 activation from within the cell indicates that CAgp130 gets JAK-associated and exists as an active dimer from its early processing stages within the ER. JAKs have been reported to act as chaperones and enhance cell surface expression for a series of receptors like MPL [26], the erythropoietin receptor EPO-R [27] or the Oncostatin M receptor OSM-R [28]. Binding of JAKs to these receptors seems to mask a negative regulatory signal, possibly an ER retention signal. In the case of CAgp130, however, this chaperone activity of JAKs is not sufficient to facilitate cell surface expression. Interestingly there is a similar study performed with a constitutive MPL mutant [7]. Mutant MPL was captured in the ER by use of the KDEL retention sequence and was shown to be associated with JAK2. However, it was not able to support factor-independent growth of transfected cells as already reported for CAgp130 [18]. Intracellular signaling was first activated by introduction of a disulfide bond and forced dimerization as it has already been reported for gp130 [29].

In order to verify whether CAgp130 at the plasma membrane activates Stat3 we utilized three neutralizing gp130 Abs [17]. B-P4 and B-T2 successfully bound surface resident CAgp130 but were insufficient in blocking its signaling activity. B-R3 does not bind to the mutated receptor. These findings are in contrast to the results of Sommer et al., who reported to block CAgp130 by the Ab B-P4 [18]. Based on our findings we conclude that the mutant receptor which localizes to the plasma membrane does not significantly contribute to constitutive Stat3 activation. In the light of these controversial experimental findings it needs to be taken into account that Abs were tested on different experimental settings and on different cell lines.

To further investigate intracellular signaling of CAgp130 we utilized dominant-negative dynamin to inhibit receptor endocytosis. If the endocytosed receptor accounts for a part of the constitutive activity as it has been shown for the EGFR (reviewed in [30]) this contribution should be omitted upon inhibition of the internalization process. Interestingly we could not detect any effect of impaired receptor endocytosis on constitutive signaling. Stat3 phosphorylation remained unaltered indicating that the endocytosed receptor does not contribute to ligand-independent activity. Our data indicating that surface bound receptor does not contribute to constitutive activity of CAgp130 are in line with already published data by Schmidt-Arras et al. [23]. However, data concerning endosomal signaling point to different directions. Given our results we come to the conclusion that endocytosed receptor does not exert any constitutive activity. On the contrary Schmidt-Arras et al. reports that endosomal signaling represents an essential part of constitutive signaling. Again there are differences in the experimental set up that could help understand this contradictory data. In our first approaches to inhibit endocytosis we also utilized the inhibitor dynasore. However, FACS analysis of treated cells revealed detrimental effects on cell viability. In a more elaborate approach we worked with dominant-negative dynamin. Our results do not rule out the possibility of endosomal signaling in the case of CAgp130. Before giving definite answers to this question the possibility has to be excluded that mutant receptor molecules can somehow circumvent classical receptor trafficking. Finally we were able to inhibit Stat3 activation emanating from CAgp130 by transfection of a dominant-negative Stat3 mutant [19]. Similarly, signaling of CAgp130 can be blocked through inhibition of JAK1 as has been recently reported [14].

## Conclusions

Newly synthesized CAgp130 is able to phosphorylate Stat3 already before reaching the cell surface. Neither neutralizing gp130 Abs nor inhibition of endocytosis is able to alter constitutive activity of the mutant receptor. These findings lead us to the conclusion that surface resident as well as endocytosed receptor do not significantly contribute to the ligand-independent and constitutive activity of CAgp130. Thus, pharmacological inhibition of CAgp130 can be most efficiently achieved by compounds that act from within the cell such as dominant-negative STAT3.

## Methods

### Materials

Restriction enzymes and Endo H (New England Biolabs, Ipswich, MA, USA), oligonucleotides (MWG-Biotech, Ebersberg, Germany), doxycycline hyclate and brefeldin A (Sigma-Aldrich), Alexa Fluor 647 conjugate of human transferrin (Invitrogen). Recombinant human IL-6 and sIL-6Rα were expressed and purified as previously described [31,32].

### Plasmid constructs

Plasmid pSVL-WTgp130-YFP [33] was digested with XhoI and BamHI and the obtained fragment was cloned into pcDNA5/FRT/TOspecial (harbors a modified MCS) resulting in the plasmid pcDNA5/FRT/TOspecial-WTgp130-YFP. For generation of CAgp130 harboring the deletion Y186-Y190 within domain D2 of gp130 fusion PCR was performed using pcDNA5/FRT/TOspecial-WTgp130-YFP as a template. In the first step two independent PCRs were performed on the sequences flanking the sequence to be deleted. Two primer-pairs were designed – one for the left and one for the right side of the deletion with complementary overhangs at the fusion site (in bold): senseP1 – 5’-AGC CTC CGG ACT CTA GCG-3’, antisenseP1 – 5’-**TTC AAT GTT AAC AAA** ATC AAC AGT GCA TGA GGT GGG-3’, senseP2 – 5’-**ACT GTT GAT** TTT GTT AAC ATT GAA GTC TGG G-3’, antisenseP2 – 5’-CCC TCT TAA ATA GGT GCG-3’. Through substitution of a single base (underlined) resulting in a silent mutation a HpaI restriction site was generated to easily distinguish CAgp130 from WTgp130 constructs. Next, the fusion PCR was performed using primers senseP1 and antisenseP2. The PCR product was first subcloned into pCR2.1-Topo. The resulting plasmid pCR2.1-Topo-CAgp130 was digested with XhoI and Asp718 and cloned into pcDNA5/FRT/TOspecial-WTgp130-YFP generating the plasmid pcDNA5/FRT/TOspecial-CAgp130-YFP.

For generation of mCherry-tagged receptor constructs mCherry-cDNA was amplified by PCR using the plasmid pcDNA5/FRT/TOspecial-Stat3-mCherry (previously constructed in our lab) as a template: senseP – 5’-CCG GTC GCG ATA TCG GTG AGC AAG GGC GAG GAG-3’, antisenseP – 5’-AGA GTC GCG GAT CCT TTA CTT GTA CAG CTC GTC C-3’. The PCR product was subcloned into pCR2.1-TOPO and the resulting plasmid was digested with EcoRV and BamHI. The generated fragment was cloned into pcDNA5/FRT/TOspecial-WTgp130-YFP resulting in the plasmid pcDNA5/FRT/TOspecial-WTgp130-mCherry. For generation of mCherry-tagged CAgp130 the fragment that resulted from XhoI and Asp718 digestion of pCR2.1-Topo-CAgp130 (see above) was cloned into pcDNA5/FRT/TOspecial-WTgp130-mCherry generating the plasmid pcDNA5/FRT/TOspecial-CAgp130-mCherry.

For generation of add-back mutants of CAgp130 previously constructed plasmids were used [13]. New constructs were generated by three-fragment-ligation. The backbone was generated by XhoI and EcoRV digestion of pcDNA5/FRT/TOspecial-WTgp130-YFP. The extracellular part of CAgp130 was isolated upon XhoI and EcoRI digestion of pcDNA5/FRT/TOspecial-CAgp130-YFP. The intracellular part of gp130 harboring mutated Tyr-residues was generated by EcoRI and EcoRV digestion of the preexisting constructs. Following constructs were generated: pcDNA5/FRT/TOspecial-CAgp130-6F-YFP, -CAgp130-Y915-YFP, -CAgp130-Y905-YFP, -CAgp130-Y814-YFP, -CAgp130-Y767-YFP, -CAgp130-Y759-YFP and -CAgp130-Y683-YFP.

For generation of the K44A dynamin construct the plasmid pMSCV-IRES-GFP (kindly provided by Dr. N. Chatain) was digested with EcoRI and SalI and the generated fragment was cloned into EcoRI and XhoI digested pcDNA3.1(+). SalI and XhoI generate complementary overhangs and upon ligation both restriction sites are destroyed resulting in the plasmid pcDNA3.1(+)-IRES-GFP. Plasmid pcDNA3.1(+)-IRES-GFP was digested with BamHI and EcoRI providing the backbone for the next cloning step. The construct pcDNA3.1(-)-HA-hu-dynamin-K44A (kindly provided by Dr. S. Wüller) was digested with BamHI and NheI to isolate the N-terminal part of HA-hu-dynamin-K44A. To generate an EcoRI site and amplify the C-terminal part of HA-hu-dynamin-K44A PCR was performed on pcDNA3.1(-)-HA-hu-dynamin-K44A: senseP – 5’-CGA GCA AGC ATA TCT TTG CC-3’, antisenseP – 5’-GCA TCG AAT TCT TAG AGG TCG AAG GGG GGC-3’. The plasmid pcDNA3.1(+)-HA-hu-dynamin-K44A-IRES-GFP was generated by three-fragment-ligation. All constructs were verified by sequencing.

### Cell culture, transient and stable transfection

HEK293 cells were grown in Dulbecco’s Modified Eagle Medium (DMEM) with Glutamax (Gibco, Germany) supplemented with 10% FCS (Lonza, Germany), 60 mg/l penicillin and 100 mg/l streptomycin (Gibco, Germany). For HEK293 cells stably expressing IL-6Rα (kindly provided by Dr. Anna Dittrich) medium was supplemented with 2 mg/l Puromycin (Invivogen, CA, USA). Transient transfections were performed with TransIT-LT-1 transfection reagent (Mirus, Madison, USA). T-REx-293 cells were stably tranfected using the Flp-In system (Invitrogen). Antibiotics for generation and maintenance of stable cell lines – blasticidin, zeocin, hygromycin B – were purchased from Invivogen.

### Preparation of cell lysates, SDS-PAGE and immunoblotting

Receptor expression was induced with 20 ng/ml or 0.5 μg/ml dox. Stimulation was performed with 200 U/ml IL-6 and 0.5 μg/ml sIL-6Rα (when needed). Upon treatment cells were washed with PBS (150 mM NaCl, 2.5 mM KCl, 8.0 mM Na_2_HPO_4_, 1.5 mM KH_2_PO_4_, adjusted to pH 7.4) and lysed with RIPA lysis buffer (50 mM Tris–HCl pH 7.4, 150 mM NaCl, 1 mM EDTA, 0.5% Nonidet P40, 1 mM NaF, 15% glycerol) supplemented with phosphatase- and protease-inhibitors (1 mM Na_3_VO_4_, 0.25 mM phenylmethylsulfonylfluoride (PMSF), 0.5 mM EDTA, 5 μg/ml aprotinin, 1 μg/ml leupeptin). Protein concentration was measured using the Bio-Rad protein assay (Bio-Rad, Germany). Lysates were subjected to SDS-PAGE, Western Blotting and immunodetection using Abs against pStat3(Y705), pStat3(S727), pStat1(Y701), pSHP2(Tyr542), pErk1/2(Thr202/Tyr204), Stat1, SHP2, Erk1/2 (Cell signaling, USA), Stat3 (BD transduction laboratories), gp130, SOCS3, dynamin, actin (Santa Cruz Biotechnology, USA), GFP (Rockland, USA) and horseradish-peroxidase conjugated secondary antibodies (DAKO, Denmark). Primary Abs were diluted 1:1000 and secondary Abs 1:2000 in TBS-N buffer (20 mM Tris–HCl pH 7.6, 140 mM NaCl, 0.1% Nonidet P40). For pTyr detection a mixture of two pTyr Abs was used: pY99 (Santa Cruz Biotechnology) (dilution 1:1000) and 4G10 (Millipore, Germany) (dilution 1:10000). Bound Abs were detected by enhanced chemiluminescence (ECL, Millipore, USA).

### Immunoprecipitation

Cell lysates were incubated o/n with 1 μg gp130 Ab M20 (Santa Cruz Biotechnology, USA). Subsequently the lysate-Ab-mixture was incubated o/n with 10 μl Dynabeads Protein G (Life Technologies, USA). Immobilized immune complexes were washed three times with RIPA lysis buffer, boiled in Laemmli buffer and subjected to SDS-PAGE and immunoblotting.

### Flow cytometry

Cells were washed with PBS, detached from the plate for 10 min with PBS/EDTA (10 mM), collected and washed with FACS buffer (PBS containing 5% FCS and 0.1% NaN_3_). Detection of surface receptor was performed with monoclonal mouse gp130 Abs – B-P8, B-P4, B-T2 and B-R3 – and followed by goat-a-mouse APC- (Dianova, Hamburg, Germany)/Alexa633-/Alexa405- (Invitrogen) conjugated secondary Abs as stated in each case. Abs against gp130 were kindly provided by Dr. J*.* Wijdenes*.* Abs were applied in a 1:100 dilution in FACS buffer. Cells were analyzed with a FACSCanto II or a LSR Fortessa (BD Biosciences). All steps were performed on ice and with ice-cold solutions.

### Confocal microscopy

Cells were grown on poly-l-lysine coated coverslips. Fixation of cells has been described previously [19]. Confocal imaging was performed with a Zeiss LSM 710 confocal microscope (Zeiss, Jena, Germany). mCherry fluorescence was detected using the 561 nm laser and a 578-696 nm bandpass filter. The cells were examined with a Zeiss LD C-apochromat 40×/1.1 water objective. Confocal images represent confocal slices of approximately 1 μm.

## Abbreviations

IHCA: Inflammatory hepatocellular adenoma; CAgp130: Constitutively active del(Y186-Y190)gp130; Dox: Doxycycline; Ab: Antibody; WB: Western blot; TCL: Total cell lysate; IP: Immunoprecipitation.

## Competing interests

The authors declare no competing of interests.

## Authors’ contributions

NR has performed most of the depicted experiments, interpreted the data and wrote the manuscript. AK and HS-V generated most of the mentioned plasmid constructs and provided technical assistance. AM generated and characterized the STAT3-Y705F-YFP expressing cells. GM-N has initiated and designed the study, interpreted the data and critically revised the manuscript. All authors have read and approved the final manuscript.

## Supplementary Material

Additional file 1 **Effect of intracellular retention of *de novo *synthesized CAgp130 on overall receptor expression.** T-REx-293-WTgp130-YFP and T-REx-293-CAgp130-YFP were left untreated or expression was induced with 20 ng/ml dox for the indicated periods of time. Cells were simultaneously treated with 100 ng/ml brefeldin A or MeOH (vehicle). Overall receptor expression was assessed by FACS analysis of the fluorescent tag. Non-induced cells (filled histograms) were used as negative controls. Click here for file

Additional file 2 **Binding of neutralizing gp130 Abs to WTgp130 and CAgp130.** T-REx-293-WTgp130-YFP (upper panel) and T-REx-293-CAgp130-YFP (lower panel) were not incubated with dox (dotted line) or expression was induced with 20 ng/ml dox for 24 h (solid line). Surface receptor was stained with gp130 Abs B-P8, B-P4, B-T2 and B-R3 and binding of primary Abs was assessed by an APC labeled secondary Ab. Non-treated cells (filled histograms) serve as negative controls. Click here for file
