# Supplementary figures and images for: Intracellular signaling prevents effective blockade of oncogenic gp130 mutants by neutralizing antibodies
Source: Cell Commun Signal. 2014 Mar 10;12:14. doi: 10.1186/1478-811X-12-14 (PMC4007646; doi:10.1186/1478-811X-12-14)

WTgp130-YFP

CAgp130-YFP

+ dox  
+ MeOH

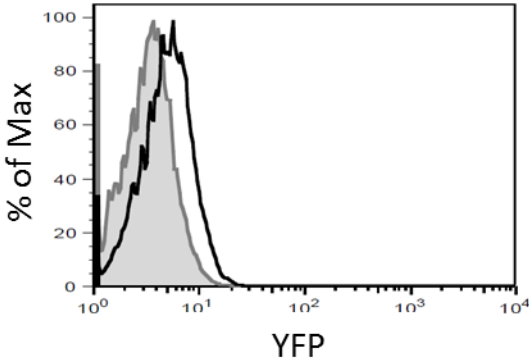

% of Max

YFP

■ - dox  
□ + 8 h treatment

+ dox  
+ brefeldin A

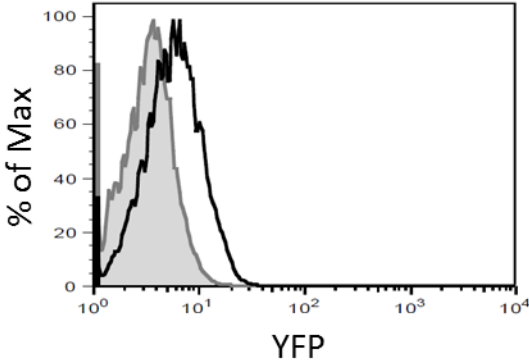

% of Max

YFP

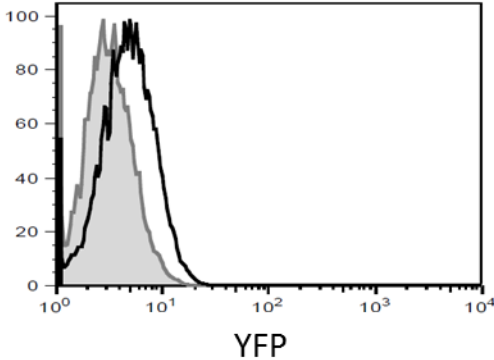

% of Max

YFP

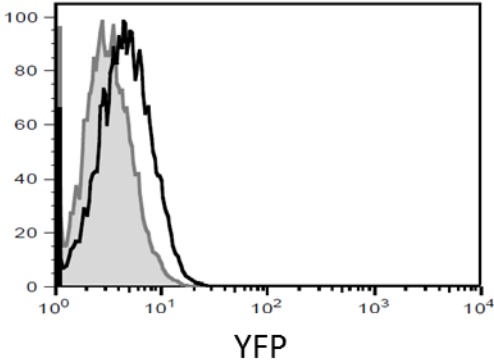

% of Max

YFP

Supplement: Additional file 1 — Effect of intracellular retention of de novo synthesized CAgp130 on overall receptor expression. T-REx-293-WTgp130-YFP and T-REx-293-CAgp130-YFP were left untreated or expression was induced with 20 ng/ml dox for the indicated periods of time. Cells were simultaneously treated with 100 ng/ml brefeldin A or MeOH (vehicle). Overall receptor expression was assessed by FACS analysis of the fluorescent tag. Non-induced cells (filled histograms) were used as negative controls. [file 1478-811X-12-14-S1.pdf]

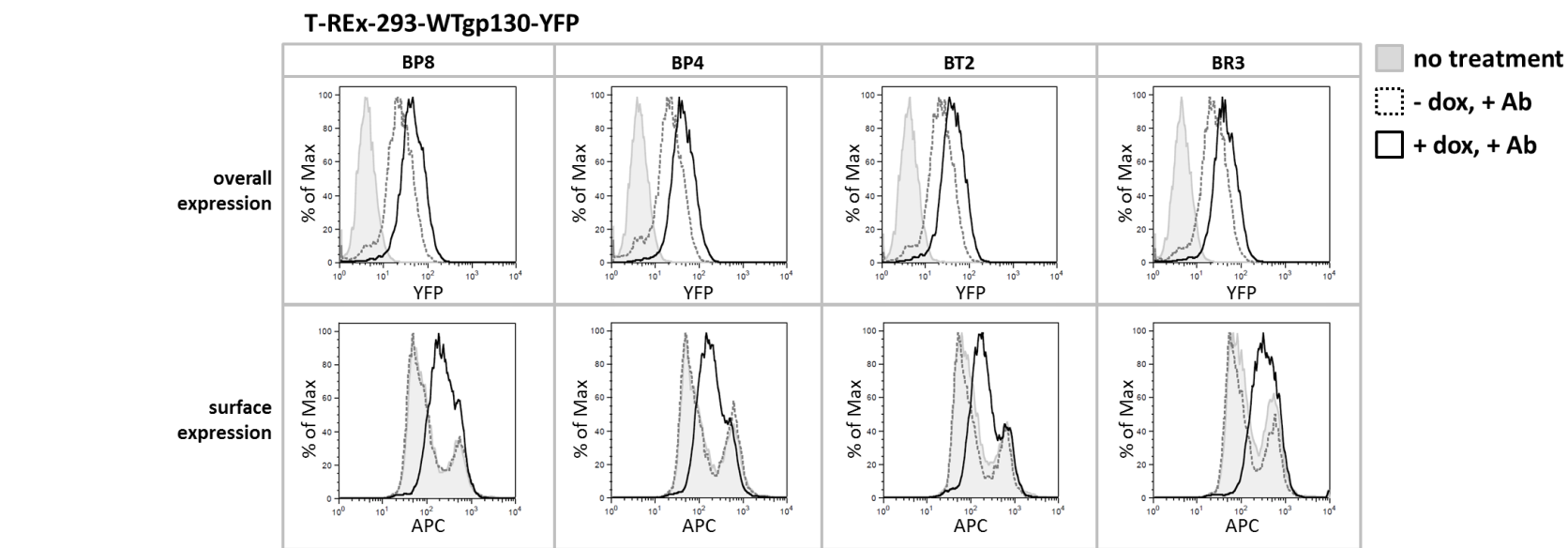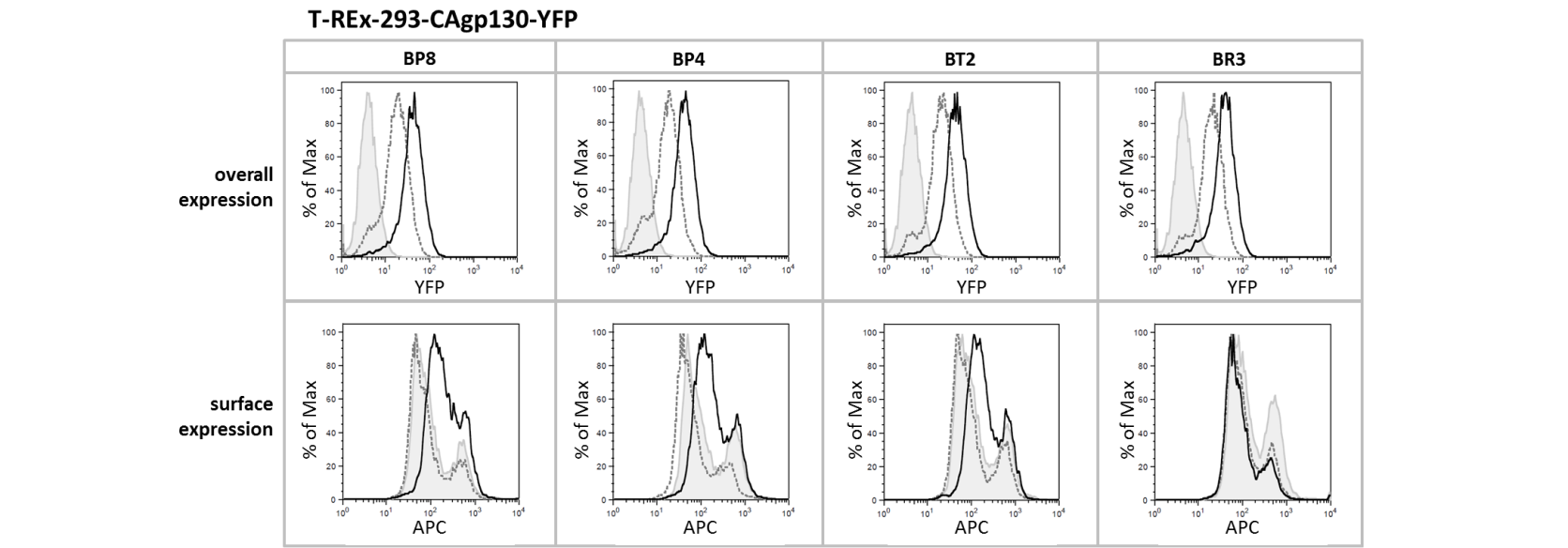

Supplement: Additional file 2 — Binding of neutralizing gp130 Abs to WTgp130 and CAgp130. T-REx-293-WTgp130-YFP (upper panel) and T-REx-293-CAgp130-YFP (lower panel) were not incubated with dox (dotted line) or expression was induced with 20 ng/ml dox for 24 h (solid line). Surface receptor was stained with gp130 Abs B-P8, B-P4, B-T2 and B-R3 and binding of primary Abs was assessed by an APC labeled secondary Ab. Non-treated cells (filled histograms) serve as negative controls. [file 1478-811X-12-14-S2.pdf]
